# Assessing the utility of bulbocavernosus reflex for predicting urological outcome in children with spinal dysraphism surgery

**DOI:** 10.1007/s00381-026-07137-8

**Published:** 2026-01-28

**Authors:** Amparo Saenz, Ivana Jankovic, Catherine Mann, Amy Lee, Divyseh Desai, Zubair Tahir, Dachling Pang, Dominic Thompson

**Affiliations:** 1 Neurosurgery Department, Great Ormond Street Hospital, London, UK; 2 Neurophysiology Department, Great Ormond Street Hospital, London, UK; 3 Urology Department, Great Ormond Street Hospital, London, UK

**Keywords:** Paediatrics, Dysraphism, Spinal, Neurophysiology, Assessment, Outcomes, Neurosurgery, Bulbocavernousus reflex

## Abstract

**Purpose:**

Surgical untethering for spinal dysraphism carries the risk of neural damage affecting sphincter control. Whilst anal sphincter motor-evoked potentials (MEP) are commonly used, they assess efferent pathways and do not directly reflect bladder innervation. The bulbocavernosus reflex (BCR) evaluates both afferent and efferent components of the sacral reflex arc, potentially serving as a better intraoperative predictor of urinary function. This study aimed to (1) assess the correlation between baseline BCR and preoperative urinary continence and (2) determine whether intraoperative BCR loss predicts postoperative urinary outcomes at one year.

**Methods:**

A retrospective observational study was conducted on children (aged 4–18 years) undergoing spinal cord untethering with intraoperative BCR monitoring. Preoperative and 1-year postoperative urological assessments included continence status, clean intermittent catheterisation (CIC) dependency, recurrent urinary tract infections (UTI), post-void residual volume (PVR), and vesicoureteral reflux (VUR). Sensitivity, specificity, and predictive values were calculated for both baseline and intraoperative BCR changes relative to urological outcomes.

**Results:**

Fifty patients met the inclusion criteria. Preoperative absence of BCR correlated with preoperative incontinence with 34.5% sensitivity and 90.5% specificity. Intraoperative BCR loss predicted postoperative incontinence with 14.8% sensitivity and 82.6% specificity. CIC dependency and recurrent UTIs were unchanged postoperatively, whilst increased PVR decreased from 20 to 12%.

**Conclusion:**

Baseline BCR has high specificity but limited sensitivity for preoperative continence. Intraoperative BCR loss is a specific but insensitive predictor of postoperative urinary dysfunction. Whilst BCR monitoring aids intraoperative decision-making, further studies are needed to optimise its role in predicting long-term bladder outcomes.

## Introduction

Inflicting damage to the neural control of the sphincters is a feared complication of surgery for spinal dysraphism. Measurement of the anal sphincter MEP is routinely used in an attempt to preserve sphincter innervation; however, it is only a proxy measure of bladder sphincter innervation; furthermore, it evaluates only the efferent component of the sphincter innervation [1].

**Fig. 1:**
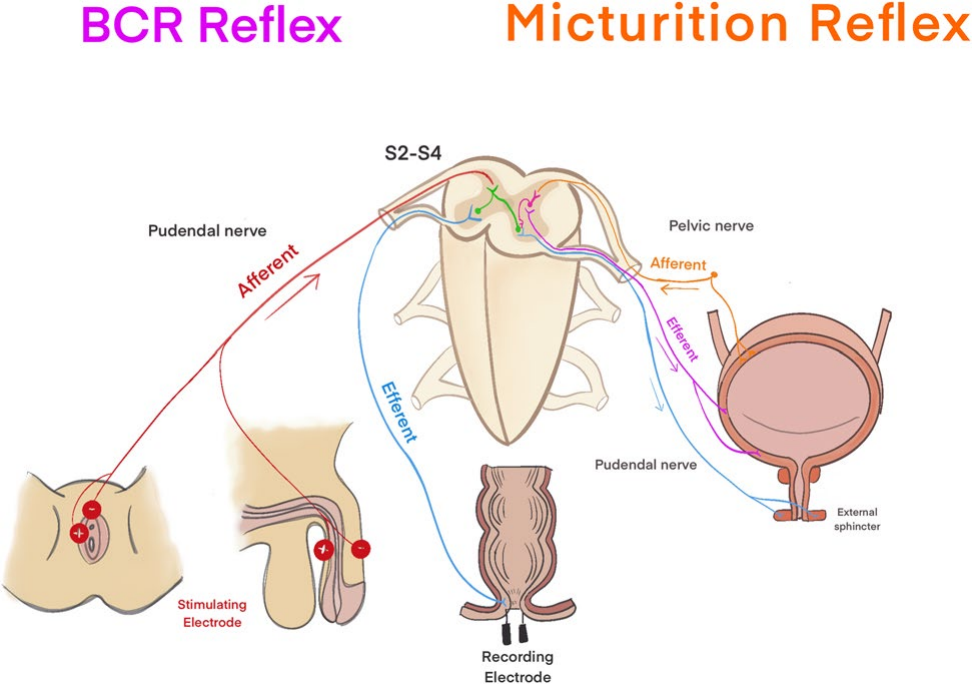
Neural pathways of the bulbocavernosus reflex (BCR) and micturition reflex. This diagram illustrates the afferent and efferent pathways of the BCR reflex (left) and micturition reflex (right), both mediated through the S2-S4 spinal segments. The BCR reflex involves stimulation of the pudendal nerve, with afferent signals (red) traveling to the spinal cord and efferent signals (blue) eliciting contraction of the external anal sphincter. The micturition reflex is regulated by the pelvic nerve, with afferent signals (orange) transmitting bladder distension to the spinal cord and efferent signals (purple and blue) coordinating detrusor contraction and external sphincter relaxation. Electrode placements for intraoperative neurophysiological monitoring are also depicted

The bulbocavernosus reflex (BCR) is an involuntary contraction of the anal sphincter in response to stimulation of the glans penis or clitoris. The anatomical basis of the BCR is an oligosynaptic reflex arc utilising afferent and efferent nerve fibres within the pudendal nerve and is mediated through the S2-S4 spinal segments [2]. Neurophysiological monitoring of the BCR permits assessment of both afferent and efferent components of the sacral reflex arc and so may be a more valuable tool to evaluate sphincter function during spinal dysraphism surgery compared with anal sphincter MEP alone [3–5]. BCR is not a direct measure of bladder innervation; however, since bladder control is also mediated through the S2-S4 sacral segments, the integrity of the BCR might act as a surrogate marker of the pathways responsible for bladder control (Fig. 1) [6–8].

Although electrophysiological monitoring of BCR is technically challenging, particularly in young children, clinical studies have confirmed the feasibility of monitoring BCR during untethering surgery [9–11]. However, correlation between BCR responses and bladder continence remains unclear.

There have been previous attempts to assess the association between intraoperative BCR and subsequent urinary incontinence after spinal dysraphism surgery. However, these studies identified notable differences in results [12, 13]. Shinjo et al. [12] reported the sensitivity of BCR loss as a predictor of incontinence to be 100%, whilst Cha et al. [13] reported a sensitivity of 35.7%. In neither of these studies did the authors comment on the correlation between preoperative urological status and baseline BCR.

The aim of this current study was twofold: first, to evaluate the sensitivity and specificity of baseline BCR in predicting preoperative urinary function in patients with spinal dysraphism undergoing untethering surgery, and second, to assess the sensitivity and specificity of maintenance/loss of BCR during surgery in predicting urinary function 12 months after surgery in the same population.

In addition to continence, we also evaluated the sensitivity and specificity of the BCR responses pre- and post-operatively for other urological outcome measures, namely, frequent urinary tract infection (UTI), increased post-void residual (PVR), and vesicoureteral reflux.

## Materials and methods

### Study design

The study design was a single-institution, retrospective observational study. This retrospective review of medical records, conducted by the medical team in charge of the patients, did not require formal ethical approval as it involved data used as part of routine clinical care. The study was conducted in accordance with the ethical standards of the 1964 Declaration of Helsinki and its later amendments.

Patients were identified through a departmental database of neurosurgical procedures performed between January 2015 and December 2021. The patient cohort comprised children between the ages of 4 and 18 undergoing spinal cord untethering procedures with intraoperative monitoring of BCR. Only children with dedicated urological assessments before and at least 1 year after surgery were included.

Demographic information and details of the underlying spinal dysraphic anomaly were identified from the electronic medical and radiological records.

Pre-operative urological assessments were conducted between 1 and 6 months before surgery.

Although the first postoperative urological assessment was carried out 3–6 months postoperatively, only assessments carried out 1 year or more after untethering surgery were used in this study to more accurately reflect urological status after recovery from any transient effects of surgery.

Urological evaluations were performed using a standardised bladder assessment comprising a urological history of voiding routine, continence, and urinary infection. Pre/post micturition bladder volumes were measured by ultrasound scans over three voids [14–18]. An ultrasound of the renal tract was performed for anatomical anomalies.

#### Incontinence

Only children older than 4 years were included to compensate for the problems of assessing continence in infants and pre-continent children. Incontinence was defined as involuntary voiding of urine ≥ two times/month during the day or night, loss of desire to urinate, or the need for intermittent urethral catheterisation [19–21].

#### Urological evaluations

##### Urinary tract infection (UTI)

Symptomatic UTI was diagnosed if there were signs and symptoms of infection like fever  ≥ 38.5 °C, associated with a positive urine culture. A positive urine culture was defined as  ≥ 104 colony-forming units/ml.

##### Clean intermittent catheterisation (CIC)

Indications for CIC comprised inability to initiate micturition, large post-void residual volume of urine (typically greater than 10% of estimated bladder capacity), recurrent urinary infection, thickening of the bladder wall, or significant vesicoureteric reflux.

##### Reduced bladder capacity

Reduced bladder capacity was defined relative to the estimated bladder capacity (EBC). A bladder capacity less than 65% of the EBC (calculated as (Age in years + 2) × 30 mL) was considered reduced.

##### Pelvicalyceal dilatation

Measurements were made by ultrasound and graded:

Mild dilation: Anterior–posterior renal pelvic diameter of 5–9 mm.

Moderate dilation: Anterior–posterior renal pelvic diameter of 10–15 mm.

Severe dilation: Anterior–posterior renal pelvic diameter greater than 15 mm.

##### Ureteral dilatation

Measurements were assessed by ultrasound and graded:

Mild dilation: Ureter diameter of 2–4 mm.

Moderate dilation: Ureter diameter of 5–7 mm.

Severe dilation: Ureter diameter greater than 7 mm.

##### Vesicoureteral reflux (VUR)

These grades are determined via a voiding cystourethrogram (VCUG).

Grade I: Reflux into the ureter only.

Grade II: Reflux into the renal pelvis without dilation.

Grade III: Mild to moderate dilation of the ureter and renal pelvis.

Grade IV: Moderate dilation and tortuosity of the ureter and renal pelvis.

Grade V: Severe dilation and tortuosity of the ureter and renal pelvis, with loss of papillary impressions.

##### Kidney atrophy

A reduction in kidney size by more than 10% compared to the expected size for the child’s age, or a kidney length below the 5th percentile for age, was considered indicative of atrophy.

#### Bulbocavernosus reflex (BCR)

A dedicated neurophysiological monitoring technician placed the electrodes and performed the BCR measurements in all cases.

Intraoperative BCR set-up was performed following total intravenous anaesthesia induction (propofol, remifentanil) and recorded with XLTEK Protektor 16 channel neuromonitoring system (XLTEK EPWorks 6.0).

Surface electrodes (Ambu® Neuroline 700) were applied to stimulate the dorsal penile and clitoral nerves. Electrodes were placed bilaterally for independent stimulation of the left and right sacral reflex arcs. For stimulation of the penile nerve, a cathode was placed on the superior lateral surface of the penis and paired with an anode placed ipsilaterally on the inferior lateral surface of the penis. For stimulation of the clitoral nerve, a cathode was placed on the surface lateral to the clitoris and paired with an anode placed ipsilaterally on the labia majora. Surface electrodes were resized depending on the surface area of stimulation and secured with cutaneous tape (Steri-Strip™, 3M Deutschland GmbH) ± cutaneous film dressing (Tegaderm™ Film, 3M Deutschland GmbH). A single train of five stimulation pulses with an interstimulus interval of 300 Hz, an intensity of 10–30 mA and a duration of 0.5 ms was delivered for all subjects. BCR potentials were recorded bilaterally (ipsilateral and contralateral to stimulation) via bilateral bipolar needle electrode recordings (Ambu® Neuroline Twisted Pair Subdermal Needle Electrodes) in the external anal sphincter at the mucocutaneous junction. A low-frequency filter was set at 30 Hz (male)/100 Hz (female), and a high-frequency filter of 2 kHz.

Baseline BCR traces were obtained post-induction and pre-incision with the patient positioned prone on the operating table. A baseline was chosen from two or more traces to demonstrate the stability and reproducibility of the waveform before surgical intervention. The presence of a BCR response was defined as a consistent, reproducible polyphasic waveform of the first oligosynaptic response (R1) of the external anal sphincter with a typical onset of > 35 ms and a minimum amplitude of 5 µV. An absent waveform was categorised when the BCR potentials were difficult to discern from the background noise (typically < 5 µV).

**Fig. 2:**
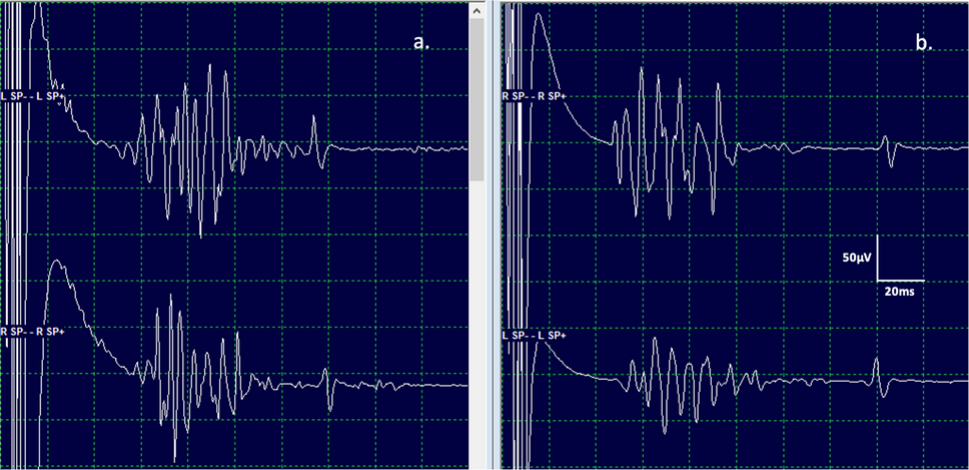
Representative bulbocavernosus reflex (BCR) waveforms. Electrophysiological recordings of ipsilateral and contralateral BCR responses following unilateral stimulation of the clitoral nerve in a 6-year-old female patient. **a** Waveforms obtained after left-sided stimulation, showing characteristic polyphasic responses in both ipsilateral and contralateral recordings. **b** Waveforms obtained after right-sided stimulation, demonstrating consistent evoked responses with similar morphology and latency. The vertical and horizontal calibration bars represent 50 µV and 20 ms, respectively. These recordings illustrate the reproducibility and integrity of the sacral reflex arc during intraoperative neuromonitoring

On opening dura, BCR waveforms were acquired regularly during the surgery and concomitantly with transcranial motor evoked potentials (periodically at the surgeon’s request due to excessive movement). A significant decrease in BCR responses was defined as a 70–80% amplitude reduction from baseline configuration with morphology change from multiphasic to monophasic, or a total absence of response despite maximising stimulation parameters. Final BCR waveforms were acquired after dural closure, and the integrity of the closure was assessed via a Valsalva manoeuvre (Fig. 2).

### Statistical analysis

Descriptive and inferential statistical analyses were conducted to assess the relationship between intraoperative bulbocavernosus reflex (BCR) responses and urological outcomes. Variables were assessed for normality using the Shapiro–Wilk test and, depending on the distribution, continuous variables, such as patient age, were summarised using means and standard deviations (SD) or median and interquartile range (IQR). Categorical variables were presented as absolute frequencies and percentages. The sensitivity, specificity, and positive and negative predictive values with 95% confidence intervals (CI) were calculated. To compare continuous variables, Student’s *t*-test or the Mann–Whitney *U* test was applied depending on data normality. Categorical variables were analysed using Fisher’s exact test or Chi^2^ test, depending on data normality. The changes in urological assessments, including CIC dependence, PVR, UTIs, and VUR, between preoperative and postoperative periods were analysed using paired *t*-tests or McNemar’s test, where appropriate.

To evaluate the diagnostic performance of preoperative BCR in predicting preoperative urinary incontinence, sensitivity, specificity, positive predictive value (PPV), and negative predictive value (NPV) were calculated with corresponding 95% confidence intervals (CI). Sensitivity was defined as the proportion of incontinent patients with absent baseline BCR, whilst specificity was the proportion of continent patients with a present BCR. PPV and NPV were computed to quantify the predictive reliability of baseline BCR in assessing urinary continence status.

The predictive value of intraoperative BCR loss for postoperative urinary incontinence at 12 months was assessed using similar diagnostic performance metrics. Additionally, the association between intraoperative BCR loss and other postoperative urological parameters—including the requirement for clean intermittent catheterisation (CIC), recurrent urinary tract infections (UTIs), increased post-void residual volume (PVR), and vesicoureteral reflux (VUR)—was evaluated using Fisher’s exact test due to the small sample size.

Significance was set at a *p*-value < 0.05 for all tests. All statistical analyses were conducted using StataCorp software (Stata, College Station, TX, USA). Given the limited sample size and event rates for certain outcomes, results were interpreted cautiously, with emphasis on confidence interval width and effect size estimation.

## Results

Ninety-nine children had undergone spinal cord untethering surgery with intraoperative BCR measurement. Thirty-five patients were excluded because they were younger than four years at the time of surgery, and 14 patients were excluded due to incomplete urological outcome data. Therefore, 50 patients satisfied the inclusion criteria.

The mean age at surgery was 9.8 years (SD 0.6), ranging from 4 to 17 years. Lipomas of the spinal cord were the commonest group (39/50 cases) and comprised filar lipoma (*n* = 20), transitional lipoma (*n* = 15), chaotic lipoma (*n* = 3), and dorsal lipoma (*n* = 1). Other dysraphic pathologies were retained medullary cord (*n* = 4), re-do myelomeningocele (*n* = 3), Currarino syndrome (*n* = 2), and limited dorsal myeloschisis (*n* = 2).

### Urological evaluation

The urological evaluations before and after surgery are summarised in Table 1.

**Table 1:** Urological evaluation and outcomes before and after surgery

Variables	Preoperative (*n* = 50)	Postoperative (*n* = 50)
Incontinence (*n* + %)	58% (29/50)	52.5% (27/50)
Requiring CIC (*n* + %)	28% (14/50)	28% (14/50)
Recurrent UTI (*n* + %)	8% (4/50)	8% (4/50)
Increase PVR (*n* + %)	20% (10/50)	12% (6/50)
Reduced capacity (*n* + %)	14% (7/50)	16% (8/50)
Pelvicalyceal dilatation (*n* + %)	14% (7/50)	14% (7/50)
Ureteral dilatation (*n* + %)	4% (2/50)	4% (2/50)
Vesicoureteral reflux (*n* + %)	6% (3/50)	4% (2/50)
Kidney atrophy (*n* + %)	2% (1/50)	2% (1/50)

*CIC* clean intermittent catheterisation, *UTI* urinary tract infection, *PVR* post-void residue

Before surgery, 29 patients had incontinence. Of these, 3 demonstrated improvement after surgery (2 following filar lipoma division and 1 after transitional lipoma excision). Among the 21 patients who were continent preoperatively, 20 remained unchanged, whilst 1 experienced a persistent deterioration following the procedure (chaotic lipoma case).

### Preoperative BCRs and urinary function

Preoperative reproducible BCRs were bilaterally documented in 35 out of 50 patients, whilst they were either absent or unrecordable on one or both sides in 15 out of 50 patients, with no significant age difference between the two groups. The absent group included transitional lipomas (6), fatty filum (2), chaotic lipoma (2), Currarino syndrome (2), and retained medullary cord (2). Based on pre-operative urological assessment, 29/50 patients were considered to have incontinence before surgery (Table 1).

Absent baseline BCRs (*n* = 15) were predictive of preoperative incontinence with a sensitivity of 34.5% (95% CI 17.9–54.3) and a specificity of 90.5% (95% CI 69.6–98.8). The overall probability that a patient was correctly classified by this diagnostic tool was 64% (95% CI 49.1–77.1) (Table 2).

**Table 2:** Sensitivity, specificity, positive and negative predictive values, positive and the accuracy of the diagnostic test with their respective 95% confidence intervals (CI)

	Preoperative absent BCR (*n*= 15) and preoperative incontinence (*n* = 29)	Loss of BCR (*n* = 8) and postoperative incontinence (*n* = 21)
% Sensitivity (95%CI)	34.5 (17.9–54.3)	14.8 (4.19–33.7)
% Specificity (95%CI)	90.5 (69.6–98.8)	82.6 (61.2–95)
% Positive predictive (95%CI) value	83.3 (51.6–97.9)	50 (15.7–84.3)
% Negative predictive value (95%CI)	50 (33.4–66.6)	45.2 (29.8–61.3)
% Diagnostic test accuracy (95%CI)	64 (49.1–77.1)	46 (31.8–60.6)

*BCR* bulbocavernosus reflex, *CI* confidence interval

### Loss of BCRs during surgery and incontinence

At the end of surgery, 8 of the 35 patients with recordable baseline BCRs demonstrated loss of responses, either unilaterally or bilaterally. None of the patients with absent BCRs preoperatively regained responses intraoperatively or postoperatively. The 8 patients with intraoperative BCR loss comprised 4 transitional lipomas, 1 dorsal lipoma, 1 redo untethering of a chaotic lipoma, and 2 cases of filum division, one of which also included resection of a presacral dermoid cyst. At the 1-year postoperative evaluation, 27 of 50 patients were incontinent. Loss of BCRs at the end of the surgery (*n* = 8) was predictive of postoperative incontinence with a sensitivity of 14.8% (95% CI 4.19–33.7) and a specificity of 82.6% (95% CI 61.2–95). The overall probability that a patient was correctly classified by this diagnostic tool was 46% (95% CI 31.8–60.6) (Table 2).

### Loss of BCRs during surgery and urological evaluations

After surgery, 14/50 patients required CIC, corresponding to the same individuals who had required it preoperatively. Loss of BCRs at the end of the surgery (*n* = 8) was predictive of postoperative need for CIC with a sensitivity of 21.4% (95% CI 4.6–50.8) and a specificity of 86.1% (95% CI 70.5–95.5).

At the 1-year evaluation, 4/50 patients experienced recurrent UTIs, the same overall number as before surgery, although not involving the same individuals. Loss of BCRs at the end of the surgery (*n* = 8) was predictive of postoperative frequent UTI with a sensitivity of 25% (95% CI0.6–80.6) and a specificity of 84.8% (95% CI71.5–93).

Before surgery, 10/50 patients had increased PVR, compared with 6/50 after surgery. Of the original 10 patients, 6 showed improvement, whilst 2 patients who had normal PVR preoperatively developed increased PVR postoperatively. Loss/maintenance of BCRs at the end of the surgery (*n* = 8) was predictive of postoperative increased PVR with a sensitivity of 33.3% (95% CI4.3–77.7) and a specificity of 86.4% (95% CI 72.6–94.8).

After surgery, 2/50 patients had VUR compared with 3/50 before surgery, representing an improvement in 1 patient. Loss/maintenance of BCRs at the end of the surgery (*n* = 8) was predictive of postoperative vesicoureteral reflux with a sensitivity of 0% (95% CI 0–84) and a specificity of 83.3% (95% CI69.8–92.5). The sensitivity and specificity for loss/maintenance of BCRs at the end of the surgery and the different urological variables are compared in Table 3.

**Table 3:** Absolute frequencies, sensitivity, and specificity with their respective 95% confidence intervals (CI) for loss/maintenance of BCRs at the end of the surgery and the different postoperative 1-year urological outcomes

Diagnostic test	Incontinence	Need for CIC	Recurrent UTI	Increased PVR	Vesicoureteral reflux
Absolute frequency	21/50	14/50	4/50	6/50	2/50
Sensitivity (95% CI)	14.3 (3.1–36.3)	21.4 (4.6–50.8)	25 (0.6–80.6)	33.3 (4.3–77.7)	0 (0–84)
Specificity (95% CI)	82.8 (64.2–94.2)	86.1 (70.5–95.5)	84.8 (71.5–93	86.4 (72.6–94.8)	83.3 (69.8–92.5)

*CIC* clean intermittent catheterisation, *UTI* urinary tract infection, *PVR* post-void residue, *CI* confidence interval

## Discussion

This study aimed to address two questions concerning intraoperative BCR monitoring: firstly, does baseline BCR correlate with preoperative urological function in children with spinal dysraphism? Secondly, is the loss of BCR during surgery predictive of the urological outcome at one year?

### BCR as a measure of urological function

The presence of BCR before surgery had a high specificity as an indicator of satisfactory urinary continence (90.5%); however, it was less accurate in identifying patients who had incontinence before surgery since the sensitivity was poor (34.5%). Only half of the patients considered to have incontinence before surgery were found to have absent baseline BCR. These findings support the contention that BCR does have a potential role as a proxy measure of the integrity of the neural pathways responsible for urinary continence. This study was limited to children older than 4 years, and it is not known at what age reliable and reproducible measurement of BCR becomes possible. Also, it is important to note that BCR measurements done here require the patient to be under anaesthesia, which may limit its feasibility in certain clinical scenarios. However, given the well-recognised prognostic limitations of urological studies to predict continence in the pre-continent child, these findings raise the possibility that if, in the future, BCR measurement could be done under light sedation before surgery, it might help neurosurgeons to distinguish cases where there is little prospect of continence (and thus little to be gained from risky surgical interventions) from those with the potential for continence but at risk of the deleterious consequences of tethering (who might, therefore, benefit from prophylactic intervention).

### BCR changes during surgery

One of the criticisms of IONM during spinal surgery is that by the time a deleterious change has been detected, it is too late, as damage has already been inflicted. With reference to lipoma surgery in particular, it is becoming increasingly recognised that more radical resection of the lipoma significantly reduces the likelihood of late deterioration due to re-tethering. However, such radical resection carries higher intraoperative risk, particularly risk to bladder function [9, 22, 23]. Preservation of the sphincter MEP during conus region surgery, whilst important, does not account for possible damage to the afferent component of the sphincteric reflex. These sensory pathways are particularly vulnerable when operating on the dorsal surface of the conus due to the close proximity of the DREZ. BCR has the potential to overcome these shortcomings. This study found that preservation of BCR during surgery correlated with postoperative urinary continence with a specificity of 82.6%. Therefore, an intact BCR response during surgery can reassure the surgeon and can be helpful in postoperative prognostication for children and families. On the other hand, loss of BCR during surgery was less accurate in predicting *failure* to attain continence after surgery, as a considerable false positive rate was still associated with the test (sensitivity of 14.8%).

Despite these limitations, we consider BCR a valuable adjunct in the electrophysiological armamentarium during untethering surgery, particularly in cases of conus region lipoma where there is now a vogue for more radical (and thus riskier) lipoma resection. In such situations, attenuation of the BCR might warn the surgeon early enough that corrective action can be taken. Like MEP, BCR is not continuously monitored but needs to be repeated throughout the procedure. From the authors’ experience, there are particularly vulnerable stages of the operation where the frequency of measurements should be increased, namely, during crotch dissection (detaching the lipoma from the dural edge), whilst resecting lipoma off the ‘white plane’, and at the time of neurulation of the placode.

### Additional analysis of CIC and other urological outcomes

The additional analysis of urological variables revealed that the need for CIC demonstrated slightly higher sensitivity (21.4%, 95% CI: 4.6–50.8) compared to incontinence (14.3%, 95% CI: 3.1–36.3), although the confidence intervals for both estimates overlap significantly. This overlap suggests substantial uncertainty around these sensitivity estimates, indicating that the true sensitivity could be similar for both outcomes. Notably, the specificity for both CIC and incontinence remained high (around 82–86%), implying that both CIC and incontinence are effective at ruling out patients who will not experience these adverse outcomes.

The analysis of additional urological variables such as recurrent UTIs, increased PVR, and vesicoureteral reflux further underscores the complexity of predicting postoperative urological outcomes using intraoperative BCR monitoring. The incidence of these outcomes was low, 8% for recurrent UTIs, 12% for increased PVR, and 4% for vesicoureteral reflux, and all had low sensitivity values coupled with wide confidence intervals, suggesting a high degree of variability. This suggests that the loss of BCR is not a reliable predictor for these specific urological outcomes. The specificity values, however, were relatively high, indicating that preservation of BCR may have a future role in identifying patients who have a lower risk of developing these urological complications. The authors acknowledge that the substantial variability reflected in the confidence intervals suggests that these measures require larger sample sizes and further investigation to determine their true predictive value.

### Pre- and postoperative BCR assessment

Two previous studies investigated the relationship between the loss of BCR during surgery and postoperative urological outcomes. Shinjo et al. [12] analysed paediatric patients undergoing surgery on the terminal spinal cord and reported a specificity for BCR of 80%, similar to the current study. However, they reported 100% sensitivity, which differs significantly from our experience. It is perhaps pertinent to note that the Shinjo study comprised only 22 patients, half of whom were less than 1 year old at the time of surgery, including three cases of myelomeningocele. Furthermore, the continence status was assessed by a neurosurgeon (not a urologist) 1 week or less after removing the urinary catheter. Their chosen urological outcome was the need for clean intermittent catheterisation (CIC), potentially excluding patients with abnormal urinary dysfunction that did not meet the threshold for CIC. By contrast, we opted for incontinence of urine as well as the need for CIC as clinical outcome measures in this study. Had we only considered patients with CIC, nearly half of those with urological problems would have been excluded from the analysis (Table 1). CIC has an essential role in protecting the upper urological tracts and facilitates social continence. However, involuntary urinary leakage in dysraphism patients who are not on CIC has a significant impact on their quality of life. Our decision to broaden the urological outcome metric was an attempt to capture the broader psychosocial as well as urological impact of bladder dysfunction.

Cha et al. [13] reported that the sensitivity and specificity of BCR for post-operative worsening of voiding function were 35.7% and 88.5%, respectively, similar to those observed in our cohort. However, in their study, the mean age was only 3.3 years, and most patients were not potty trained; the outcomes measures were according to urodynamic data only (absence of detrusor contraction during voiding, dyssynergia, voiding with reduced bladder compliance, and a widely open bladder neck with leakage during the filling phase).

In addition to correlating BCR with functional outcomes of bladder function, the current study differs from others in that it correlates preoperative urological assessment with baseline BCR measurements at the commencement of surgery. This additional evaluation allowed us to better understand the relationship between BCR and urological function before the confounding factor of surgical intervention.

In summary, this study contributes to the growing body of literature regarding the utility of BCR monitoring during spinal dysraphism surgery in children and its prognostic value for urological status. Although previous studies have attested to the specificity and sensitivity of BCR monitoring [12, 13], this study builds upon these findings by examining the preoperative correlation between BCR and urological function. By doing so, we provide a more comprehensive understanding of the potential benefits and limitations of BCR testing after spinal dysraphism surgery.

### Limitations

In young children, bladder voiding patterns can be highly variable, and accurate diagnosis of early neurogenic bladder dysfunction is problematic. In this study, we used a crude measure, ‘incontinence’, as the primary urological outcome measure because it holds the highest clinical significance for parents and clinicians. However, the limitations of such a broad term must be considered when evaluating the results.

The authors acknowledge the heterogeneity of the dysraphic disorders in this series but emphasise that this study was *not* intended to discriminate between the urological prognosis of differing dysraphic anomalies but rather to assess the utility of BCR in assessing urological function.

The small patient population and the proportionately low rate of BCR worsening during surgery are factors that compromise the power of this study.

A further limitation is the choice of the 1-year postoperative endpoint. Whilst this time frame was chosen to allow recovery from surgery and avoid confounding from transient dysfunction, urinary function may also deteriorate within this period due to progressive tethered cord syndrome. This possibility could reduce the reliability of BCR as a predictor of long-term outcome.

Finally, it is acknowledged that reliable BCR responses could only be detected in 70% of cases in this series; in the experience of Sala et al., the figure was 59% [24]. Further work is required to understand how electrophysiological evaluation of BCR can be optimised.

## Conclusion

BCR is a valuable tool in the surgical armamentarium when performing untethering surgery in children; however, as with all IONM techniques, it has limitations and requires experience to perform and interpret. Recordable BCR at the commencement of surgery correlates with preoperative clinical assessment of urinary continence, raising the possibility that BCR could become part of initial clinical assessment and risk stratification for children with complex dysraphism. The maintenance of BCR at the end of the surgery has high specificity for urological continence 12 months after surgery. Surgeons should diligently pay attention to BCR responses when operating on complex dysraphic anomalies, particularly during radical resection of lumbosacral lipoma.

## Data Availability

No datasets were generated or analysed during the current study.

## References

[1] Pang D, Casey K (1983) Use of an anal sphincter pressure monitor during operations on the sacral spinal cord and nerve roots. Neurosurgery 13:562–568 6358936 10.1227/00006123-198311000-00013

[2] Skinner SA, Vodušek DB (2014) Intraoperative recording of the bulbocavernosus reflex. J Clin Neurophysiol 31:313–322 25083842 10.1097/WNP.0000000000000054

[3] Overzet K, Jahangiri FR, Funk R (2020) Bulbocavernosus reflex monitoring during intramedullary conus tumor surgery. Cureus 12:e7233 32280574 10.7759/cureus.7233 PMC7145379

[4] Kim K (2021) Intraoperative neurophysiology monitoring for spinal dysraphism. J Korean Neurosurg Soc 64:143–150 32905697 10.3340/jkns.2020.0124 PMC7969044

[5] Morota N (2019) Intraoperative neurophysiological monitoring of the bulbocavernosus reflex during surgery for conus spinal lipoma: what are the warning criteria? J Neurosurg Pediatr. 10.3171/2018.12.PEDS18535 30797211 10.3171/2018.12.PEDS18535

[6] Vodusek DB, Janko M (1990) The bulbocavernosus reflex. A single motor neuron study. Brain 113(Pt 3):813–820 2364270 10.1093/brain/113.3.813

[7] Fan X, Li K, Liu J et al (2024) The predictive value of intraoperative bulbocavernosus reflex monitoring for postoperative voiding function in patients with conus medullaris and cauda equina tumors: a retrospective single center study. Spine J 24:2314–2321 39154940 10.1016/j.spinee.2024.08.019

[8] Choi J, Kim J-S, Hyun S-J et al (2022) Efficacy of intraoperative bulbocavernosus reflex monitoring for the prediction of postoperative voiding function in adult patients with lumbosacral spinal tumor. J Clin Monit Comput 36:493–499 33682080 10.1007/s10877-021-00678-0

[9] De Vloo P, Sharma J, Alderson L et al (2022) Radical resection of lumbosacral lipomas in children: the Great Ormond Street Hospital experience. Childs Nerv Syst 38:1113–1123 35262755 10.1007/s00381-022-05483-x

[10] Valentini LG, Selvaggio G, Erbetta A et al (2013) Occult spinal dysraphism: lessons learned by retrospective analysis of 149 surgical cases about natural history, surgical indications, urodynamic testing, and intraoperative neurophysiological monitoring. Childs Nerv Syst 29:1657–1669 24013336 10.1007/s00381-013-2186-5

[11] Xu K, He J, Wang L (2022) A systematic review and meta-analysis of minimally invasive surgery in children with occult tethered cord syndrome. Transl Pediatr 11:403–410 35378968 10.21037/tp-22-72 PMC8976679

[12] Shinjo T, Hayashi H, Takatani T et al (2019) Intraoperative feasibility of bulbocavernosus reflex monitoring during untethering surgery in infants and children. J Clin Monit Comput 33:155–163 29520678 10.1007/s10877-018-0127-2

[13] Cha S, Wang K-C, Park K et al (2018) Predictive value of intraoperative bulbocavernosus reflex during untethering surgery for post-operative voiding function. Clin Neurophysiol 129:2594–2601 30448714 10.1016/j.clinph.2018.09.026

[14] Ballstaedt L, Leslie SW, Woodbury B. Bladder Post Void Residual Volume. 2024 Feb 28. In: StatPearls [Internet]. Treasure Island (FL): StatPearls Publishing; 2025 Jan. https://europepmc.org/article/nbk/nbk539839. Accessed 14 Oct 2022

[15] Paliwalla M, Park K (2014) A practical guide to urinary tract ultrasound in a child: pearls and pitfalls. Ultrasound 22:213–222 27433222 10.1177/1742271X14549795 PMC4760558

[16] Hashim H, Blanker MH, Drake MJ et al (2019) International Continence Society (ICS) report on the terminology for nocturia and nocturnal lower urinary tract function. Neurourol Urodyn 38:499–508 30644584 10.1002/nau.23917

[17] Craig JC, Simpson JM, Williams GJ et al (2009) Antibiotic prophylaxis and recurrent urinary tract infection in children. N Engl J Med 361:1748–1759 19864673 10.1056/NEJMoa0902295

[18] Kaefer M, Zurakowski D, Bauer SB et al (1997) Estimating normal bladder capacity in children. J Urol 158:2261–2264 9366371 10.1016/s0022-5347(01)68230-2

[19] Craig JC, Simpson JM, Williams GJ, Lowe A, Reynolds GJ, McTaggart SJ, Hodson EM, Carapetis JR, Cranswick NE, Smith G, Irwig LM, Caldwell PH, Hamilton S, Roy LP; Prevention of Recurrent Urinary Tract Infection in Children with Vesicoureteric Reflux and Normal Renal Tracts (PRIVENT) Investigators. Antibiotic prophylaxis and recurrent urinary tract infection in children. N Engl J Med. 2009 Oct 29;361(18):1748–59. 10.1056/NEJMoa0902295 10.1056/NEJMoa0902295 19864673

[20] Austin PF, Bauer SB, Bower W et al (2016) The standardization of terminology of lower urinary tract function in children and adolescents: update report from the standardization committee of the International Children’s Continence Society. Neurourol Urodyn 35:471–481 25772695 10.1002/nau.22751

[21] Newman DK, Willson MM (2011) Review of intermittent catheterization and current best practices. Urol Nurs 31:12–28, 48; quiz 29 21542441

[22] Pang D, Zovickian J, Oviedo A (2009) Long-term outcome of total and near-total resection of spinal cord lipomas and radical reconstruction of the neural placode: part I-surgical technique. Neurosurgery 65:511–28; discussion 528–9 10.1227/01.NEU.0000350879.02128.80 19687697

[23] La Marca F, Grant JA, Tomita T, McLone DG (1997) Spinal lipomas in children: outcome of 270 procedures. Pediatr Neurosurg 26:8–16 9361112 10.1159/000121155

[24] Sala F, Manganotti P, Grossauer S et al (2010) Intraoperative neurophysiology of the motor system in children: a tailored approach. Childs Nerv Syst 26:473–490 20145936 10.1007/s00381-009-1081-6

